# Clinical and MRI features contributing to the clinico-radiological dissociation in a large cohort of people with multiple sclerosis

**DOI:** 10.1007/s00415-025-12977-6

**Published:** 2025-04-09

**Authors:** Abhineet Ojha, Silvia Tommasin, Claudia Piervincenzi, Viola Baione, Emma Gangemi, Antonio Gallo, Alessandro d’Ambrosio, Manuela Altieri, Nicola De Stefano, Rosa Cortese, Paola Valsasina, Nicolò Tedone, Carlo Pozzilli, Maria A. Rocca, Massimo Filippi, Patrizia Pantano, The INNI Network, Abhineet Ojha, Nikolaos Petsas, Costanza Giannì, Loredana Storelli, Stefania Sala, Elisabetta Pagani, Paolo Preziosa Alvino Bisecco, Riccardo Borgo, Valentina Rippa, Fabrizio Esposito

**Affiliations:** 1 Department of Human Neurosciences, Sapienza University of Rome, Rome, Italy; 2 Unicamillus-Saint Camillus International University of Health Sciences, Rome, Italy; 3 Department of Medicine, Surgery and Neuroscience, University of Siena, Siena, Italy; 4 Department of Advanced Medical and Surgical Sciences, 3t MRI‑Center, University of Campania “Luigi Vanvitelli”, Naples, Italy; 5 Neuroimaging Research Unit, Division of Neuroscience, IRCCS San Raffaele Scientific Institute, Milan, Italy; 6 Neurology Unit, IRCCS San Raffaele Scientific Institute, Milan, Italy; 7 Vita-Salute San Raffaele University, Milan, Italy; 8 Neurorehabilitation Unit, IRCCS San Raffaele Scientific Institute, Milan, Italy; 9 Neurophysiology Service, IRCCS San Raffaele Scientific Institute, Milan, Italy; 10 IRCSS NEUROMED, Pozzilli, Italy

**Keywords:** Multiple Sclerosis, Clinico-radiological dissociation, Magnetic resonance imaging (MRI)

## Abstract

**Background:**

People with Multiple Sclerosis (PwMS) often show a mismatch between disability and T2-hyperintense white matter (WM) lesion volume (LV), that in general is referred to as the clinico-radiological paradox.

**Objectives:**

This study aimed to understand how an extensive clinical, neuropsychological, and MRI analysis could better elucidate the clinico-radiological dissociation in a large cohort of PwMS.

**Methods:**

Clinical scores, such as Expanded Disability Status Scale (EDSS), 9 Hole Peg Test (9HPT), 25-foot Walking Test (25-FWT), Paced Auditory Serial Addition Test at 3 s (PASAT3), Symbol digit Modalities Test (SDMT), demographics, and 3 T-MRI of 717 PwMS and 284 healthy subjects (HS) were downloaded from the INNI database. Considering medians of LV and EDSS scores, PwMS were divided into four groups: low LV and disability (LL/LD); high LV and low disability (HL/LD); low LV and high disability (LL/HD); high LV and disability (HL/HD). MRI measures included: volumes of gray matter (GM), WM, cerebellum, basal ganglia and thalamus, spinal cord (SC) area, and functional connectivity of resting-state networks.

**Results:**

The clinico-radiological dissociation involved 36% of our sample. HL/LD showed worse SDMT scores and lower global and deep GM volumes than HS and LL/LD. LL/HD showed lower GM, thalamus, and cerebellum volumes, and SC area than HS, and lower SC area than LL/LD.

**Conclusions:**

A more extensive clinical assessment, including cognitive tests, and MRI evaluation including deep GM and SC, could better describe the real status of the disease and help clinicians in early and tailored treatment in PwMS.

**Supplementary Information:**

The online version contains supplementary material available at 10.1007/s00415-025-12977-6.

## Introduction

Multiple Sclerosis (MS) is a chronic inflammatory and degenerative disease of the central nervous system (CNS), characterized by demyelination, axonal loss, and neurodegeneration, whose highly variable course is periodically evaluated by clinical and Magnetic Resonance Imaging (MRI) exams.

Disability assessment in people with Multiple Sclerosis (PwMS) typically lies on the Expanded Disability Status Scale (EDSS) [1], despite its known limitations [2, 3]. Indeed, the use of EDSS has several caveats, such as the strong influence of ambulation, the underestimation of cognitive and upper limb impairment, and a high intra- and inter-rater variability [4].

On the other hand, use of conventional MRI for monitoring MS through the evaluation of white matter (WM) lesions cannot fully explain the complexity of MS pathology and provide complete information on the extent of MS damage, due to the histopathological non-specificity of T2-weighted sequences [5].

Therefore, conventional MRI measures and disability assessment by EDSS are poorly correlated [6]. Moreover, some PwMS exhibit a mismatch between disability and WM lesion burden, the so-called “clinico-radiological paradox” [7]. Indeed, we can observe PwMS with extensive involvement of WM and a low EDSS score, as opposed to PwMS showing a disability which is worse than expected by the extent of MRI T2-hyperintense lesions.

Since its first description [7], many efforts were directed toward the explanation of the clinico-radiological paradox by considering possible confounders, e.g., the specific pattern of spatial distribution of WM lesions, brain atrophy, and spinal cord damage, as well as underestimation of some clinical domains, e.g., cognitive dysfunction. Using more specific and comprehensive clinical scales [8–10] and/or MRI measures [8, 10, 11], also including functional MRI [12], a better correlation between clinical impairment and CNS damage has been described, leading to question the existence of this paradox [13, 14]. However, periodical evaluation of PwMS in a clinical setting generally does not rely on more accurate but time-consuming tools, as clinical scales assessing disability in all domains of human activities and advanced MRI sequences requiring post-processing analysis.

Understanding the clinico-radiological paradox could improve our knowledge of the pathophysiological mechanisms underlying disability in MS and, in turn, could guide the therapeutic approach. We hypothesized that different MRI factors, variously associated with each other, may contribute to generating the two main patterns in this paradox: in PwMS with high lesion burden and low disability, adaptive plasticity could play a predominant role in preserving motor ability, while in PwMS with low lesion load and high disability, underestimating structural damage, especially in the spinal cord, may be of primary importance. In addition, the underestimation of some clinical features, not captured by EDSS, e.g., the cognitive status, may contribute to this dissociation in PwMS with high lesion load and low disability. Therefore, we aimed to better understand factors contributing to the clinico-radiological paradox through an extensive and systematic analysis of multiple clinical measures and MRI parameters causing a clinico-radiological dissociation in a large and well-characterized series of PwMS.

## Materials and methods

### Population

Clinical/neuropsychological data, demographics, and MRI of PwMS and healthy subjects (HS) were retrospectively retrieved from the Italian Neuroimaging Network Initiative (INNI) repository (https://database.inni-ms.org [15]). Study protocols were approved by the local ethics committees and have been performed in accordance with the ethical standards laid down in the 1964 Declaration of Helsinki and its later amendments. Both PwMS and HS signed a written informed consent form. To protect subjects’ privacy, all data were anonymized.

Inclusion criteria in the INNI database are reported elsewhere [15].

To be included in this study, PwMS had to satisfy the following criteria: availability of demographic data (age, sex, and years of education), clinical information (disease duration and phenotype), and EDSS score; availability of anatomical three‐dimensional T1‐weighted images (3DT1) and resting-state functional MRI (RS-fMRI); availability of T2-hyperintense WM lesion volume (LV) maps.

The availability of demographic data, and both 3DT1 and RS-fMRI scans were also required for HS. HS were further selected to be age- and sex-matched with PwMS.

Other clinical and neuropsychological scales, including nine-hole peg test (9-HPT), timed 25-foot walking test (25-FWT), paced auditory serial addition test 3 s (PASAT3), and symbol digit modalities test (SDMT), were also available in the database. PASAT3 and SDMT were corrected by education and standardized to a sample of healthy controls following Amato et al. 2006 [16] and the number of PwMS and HS who scored less than the cutoff was evaluated for both the tests.

### Identification of clinical/MRI dissociation groups

To investigate the dissociation between MRI lesion burden and clinical disability, PwMS were divided into four groups according to the median of LV and EDSS: low LV and low disability (LL/LD); high LV and low disability (HL/LD); low LV and high disability (LL/HD); high LV and high disability (HL/HD).

### MRI data acquisition

MRI scans were acquired using four 3.0 T scanners. MRI sequences included 3DT1, proton‐density/T2‐weighted and/or Fluid Attenuated Inversion Recovery (FLAIR) images, and RS-fMRI. Details regarding acquisition protocols are reported in Supplementary materials (see Supplementary Table 1).

### MRI analysis

#### Data pre-processing

Pre-processing and post-processing of structural and functional images were performed using fMRIPrep 20.2.3 [17] and FSL v6.0.0 (https://fsl.fmrib.ox.ac.uk/fsl/docs/#/). The structural and functional pipelines are described in the Supplementary Materials.

### Structural MRI

Structural measures included WM LV, total intracranial volume (TIV), GM, WM, deep GM (thalamus, caudate nucleus, putamen, and globus pallidus) and cerebellum volumes, and spinal cord (SC) area.

#### White matter lesion volume

T2-hyperintense lesions were segmented at each site on either proton-density or FLAIR images using a semi-automated technique (Jim, Xinapse System, Colchester, UK; http://www.xinapse.com). WM lesion volume was calculated using the FSL toolbox. To avoid center-dependent variability of the lesion marking, all lesion masks were further checked by experts.

#### Global brain volumes

To improve tissue segmentation, 3DT1 were lesion-filled with the tool available in the FMRIB Software Library (FSL, version 6.0.0) [18]. TIV, GM, and WM volumes were calculated from lesion-filled 3DT1 images using SPM CAT12 (http://www.neuro.uni-jena.de/cat/).

#### Deep gray matter

Deep GM structures, i.e., the thalamus, caudate nucleus, putamen, and globus pallidus, were segmented from 3DT1 images using FMRIB’s Integrated Registration and Segmentation Tool (FIRST, http://fsl.fmrib.ox.ac.uk/fsl/fslwiki/FIRST) [19].

For each deep GM structure, the left and right volumes were summed.

#### Cerebellum volume

Cerebellum volume was calculated using the spatially unbiased infratentorial template toolbox (SUIT), version 3.3 (http://www.diedrichsenlab.org/imaging.suit.htm), implemented in SPM12 (http://www.fil.ion.ucl.ac.uk/spm) [20]. Each subject’s cerebellum was isolated and cropped from the 3DT1 anatomical images. Each cropped image was subsequently normalized into SUIT space using the affine transformation matrix and non-linear flow field. Each cerebellum was, therefore, resliced in the atlas space, modulating to grant volume preservation. Lastly, the probabilistic cerebellar atlas was resliced back into the individual subject space. Cerebellar structures included ten bilateral regions of the cerebellum, i.e., lobules I-IV, V, VI, Crus I, Crus II, VIIb, VIIIa, VIIIb, IX, and X.

To account for head size variability, both global and regional brain volumes, i.e., GM, WM, deep GM, and cerebellum, were normalized to the TIV.

#### Spinal cord C2–C3 area

SC area was calculated by applying the Spinal Cord Toolbox version 0.5.0.1 (SCT) [21] on the bias-corrected 3DT1 at the level of C2–C3. Segmentation of C2–C3 was obtained through the sct_propseg algorithm, which uses a machine learning-based method (OptiC) to automatically detect the approximate center of the spinal cord [22] from where a mesh is propagated to recreate the SC. After segmentation, vertebrae were labeled using the sct_label_vertebrae tool by matching with the PAM50 template [23] and two expert observers checked the posterior tip of the C2–C3 intervertebral disk to ensure the correct labeling. The automated vertebral labeling step failed in 58 cases, and in these cases, manual labeling of the posterior tip of the intervertebral disks C1–C2, C2–C3, and C3–C4 was performed using SCT's sct_label_utils. Finally, the SC area was calculated using the sct_process_segmentation tool as the average area of the C2–C3 level.

### Functional MRI

Functional (f)MRI pre-processing included skull stripping, slice time correction, motion correction, physiological noise correction, 6-mm spatial smoothing, and co-registration of the functional and anatomical scans, followed by registration onto the MNI standard template (further details in the supplementary material). The fMRIs were then corrected from the signal of the WM and cerebrospinal fluid, [0.01–0.1] Hz band-pass filtered, and the first volumes (12 s) were removed to ensure that the data reached a steady state.

#### Independent component analysis of functional MRI

For group-wise independent component analysis (ICA), a single four-dimensional (4D) dataset was created by temporally concatenating pre-processed functional data. The dimensionality of group ICA was performed using 20 components [24]. To reduce the number of comparisons, six resting-state networks (RSNs) of interest were identified via spatial correlation coefficients (fslcc tool) using RSNs generated by Smith et al. [24] and Yeo et al. [25] as templates, and then verified by expert visual inspection. Three RSNs were related to the motor domain, i.e., the sensorimotor (SMN), basal ganglia (BGN), and cerebellum (CBN) networks, and the other three RSNs were related to the cognitive domain, i.e., default mode (DMN), and right and left frontoparietal (RFN, LFN) networks. The set of spatial maps from the group average analysis was used to generate subject-specific versions of the spatial maps and associated time series using a dual regression technique [26, 27]. For each subject, the group average set of spatial maps was first regressed (as spatial regressors in a multiple regression) into the subject’s 4D space–time dataset, resulting in a set of subject-specific time series, one per group-level spatial map. These time series were then regressed (as temporal regressors in a multiple regression) into the same 4D dataset, resulting in a set of subject-specific spatial maps, one per group-level spatial map.

### Feature harmonization

To remove the effects due to scanner variability, we applied the ComBat harmonization [28] to all volumetric data, i.e., GM, WM, deep GM and cerebellum volumes, and to SC area. Resting-state functional connectivity (RS-FC) was harmonized separately in each of the six networks, following the approach of Fortin et al. [28].

Age and sex were included as biological covariates in the harmonization process to prevent their distributions among sites from being mistaken for scanner-related effects.

### Statistics

Statistical analysis was performed with the R software version 4.1.2 (R Foundation for Statistical Computing, Vienna, Austria. URL: https://www.R-project.org/). Pearson's chi-squared test (Chi-square test) was used to test for differences in sex and phenotype, as well as in frequency of scores less than normative cutoff in PASAT3 and SDMT performances, among groups, i.e., LL/LD, LL/HD, HL/LD, HL/HD, and HS. The analysis of variance (ANOVA) was used to test for differences in age among groups. ANCOVA was used to investigate group differences in volumetric data and SC area, including age, sex, and TIV as covariates of no interest. The Tukey test was used as a post hoc test. Significance was stated if *p* < 0.05 after adjusting for multiple comparisons.

For fMRI data, we utilized voxel-wise ANCOVA and FSL randomize (5000 permutations, significance at *p* < 0.05 after false discovery rate, FDR, correction, and cluster size larger than 100 voxels) for non-parametric between-group comparisons. Age, sex, and TIV were included as covariates of no interest.

## Results

Data of 717 PwMS and 284 age- and sex-matched HS were downloaded from the INNI database. Out of them, 605 PwMS and 192 HS were examined with the 9-HPT, 525 PwMS and 160 HS with the 25-FWT, 616 PwMS and 157 HS with the PASAT3, and 479 PwMS and 83 HS with the SDMT. EDSS was available for all PwMS.

We obtained SC area measurements from 696 subjects; 214 MS and 91v HS were rejected due to the absence of C3 vertebrae in the 3DT1 field of view. All remaining MRI measures were available for all PwMS and HS.

PwMS had a median EDSS = 2 and a median T2-hyperintense LV = 4.76 ml. Following the EDSS and LV median-based group selection, 242 PwMS were assigned to LL/LD, 138 to HL/LD, 121 to LL/HD, and 216 to HL/HD group. The total number of PwMS showing a dissociation between LV and EDSS (groups HL/LD and LL/HD) was 259, representing 36% of the entire patient sample.

**Fig. 1:**
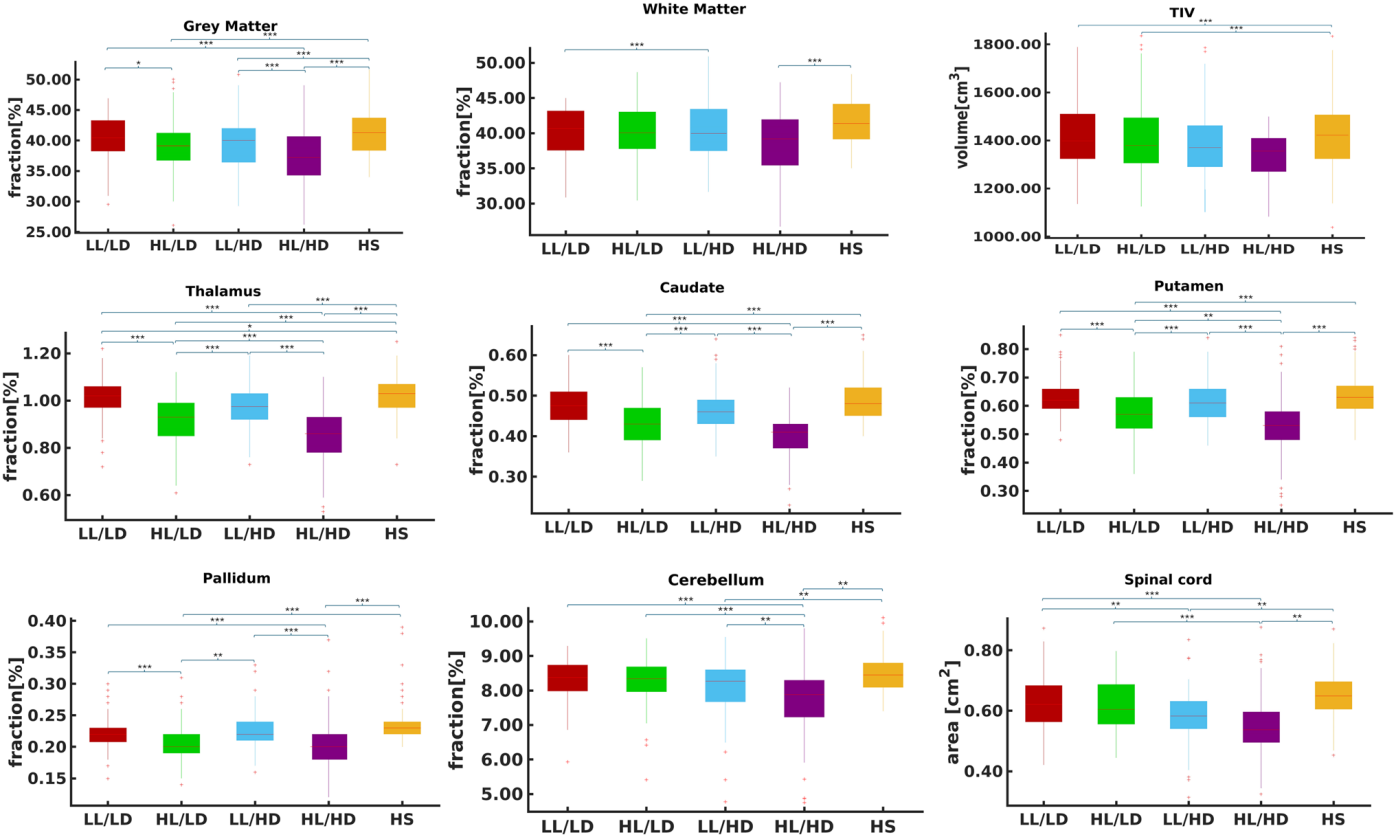
Boxplot showing global and regional brain volumes normalized to total intracranial volume (fraction %) and spinal cord area (cm2) in the four groups of people with multiple sclerosis (PwMS) and healthy controls (HS). Significant differences are reported as ***(*p* < 0.001), ** (*p* < 0.01), and * (*p* < 0.05), after multiple comparison corrections

**Fig. 2:**
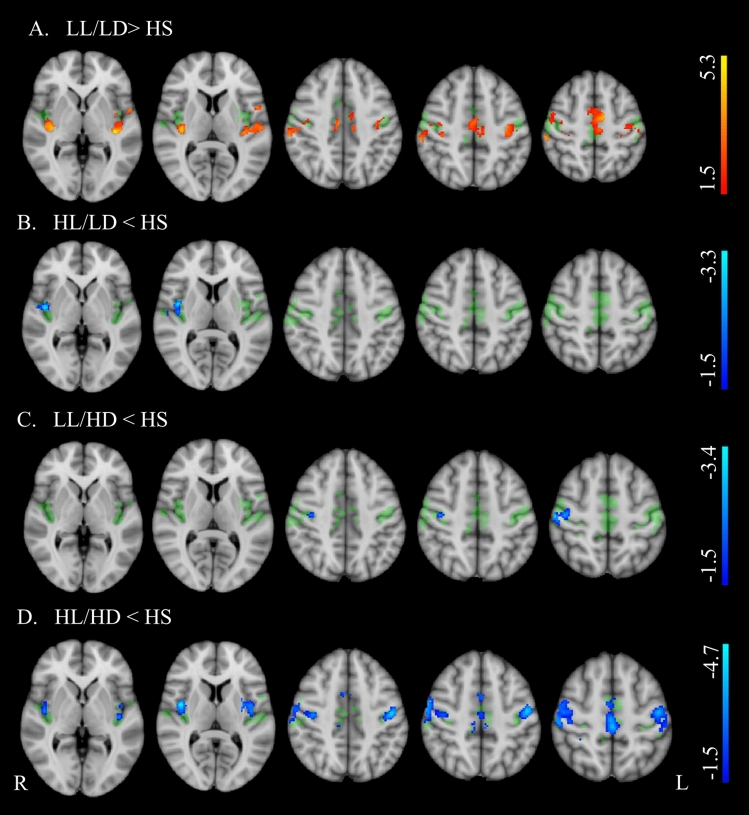
Group differences in resting-state functional connectivity between people with multiple sclerosis (PwMS) and healthy controls (HS) in the sensorimotor network (SMN). Results (*p* < 0.05, FDR corrected) are overlaid onto the F map (green) in the MNI152 standard brain. The red-yellow color indicates areas of higher functional connectivity (FC), while the blue–light blue color indicates areas of lower FC in PwMS than healthy controls. The color bars represent *t* values

**Fig. 3:**
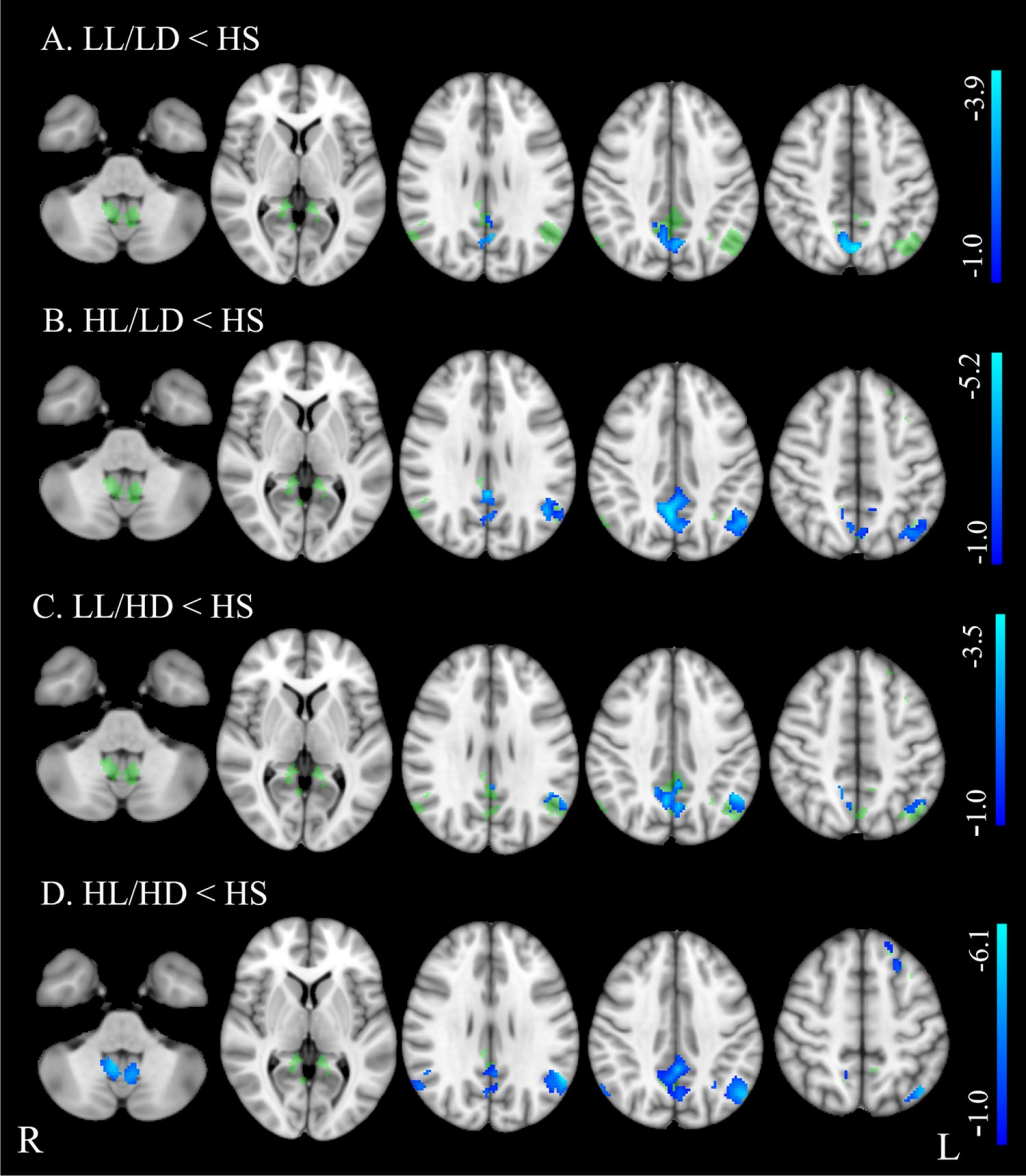
Group differences in resting-state functional connectivity between people with multiple sclerosis (PwMS) and healthy controls (HS) in the default mode network (DMN). Results (*p* < 0.05, FDR corrected) are overlaid onto the F map (green) in the MNI152 standard brain. Blue–light blue indicates areas of lower functional connectivity (FC) in PwMS than healthy controls. The color bars represent *t* values

**Fig. 4:**
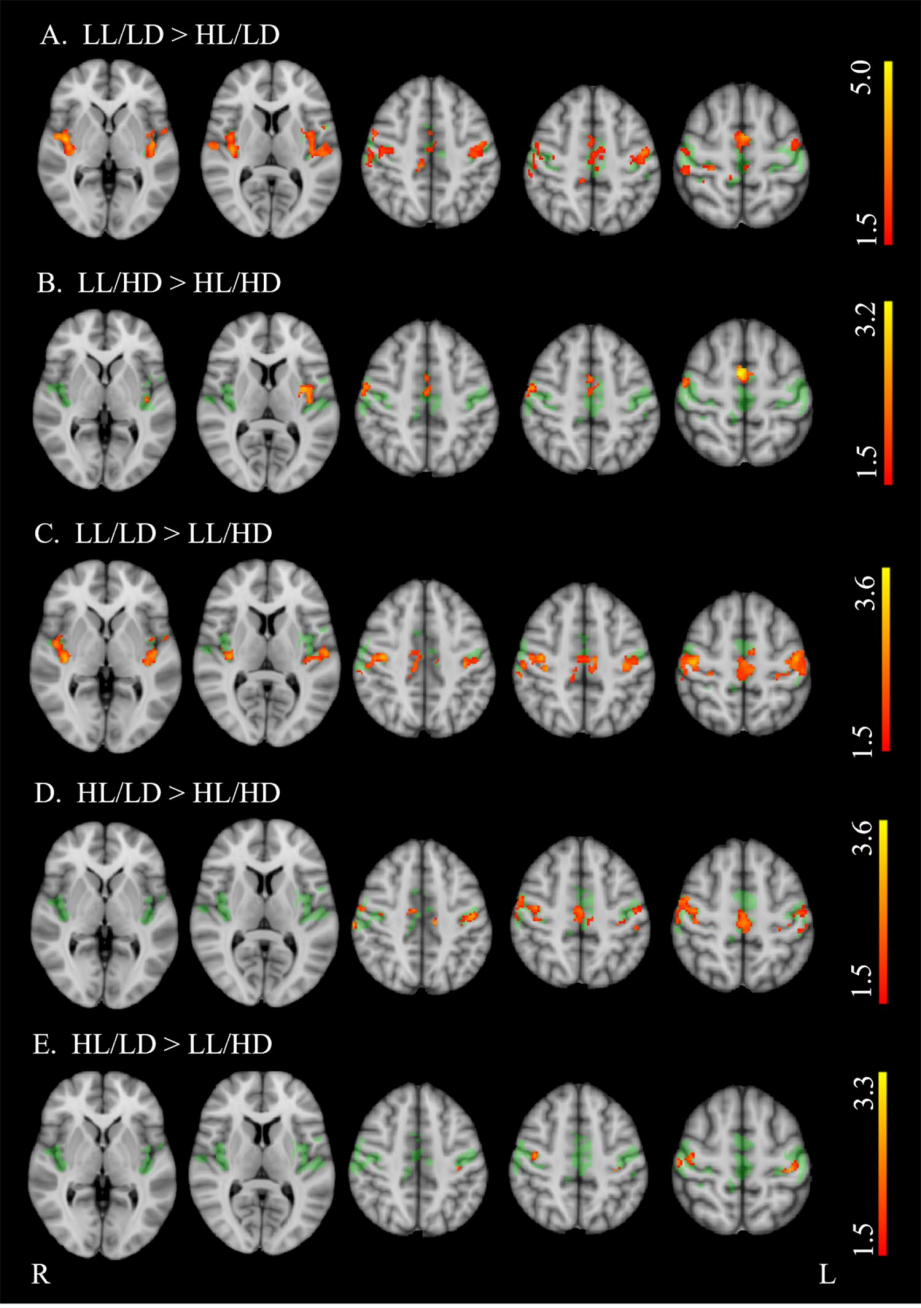
Group differences in resting-state functional connectivity among four groups of people with multiple sclerosis (PwMS) in the sensorimotor network (SMN). *LL/LD* low lesion volume and low disability, *HL/LD* high lesion volume and low disability, *LL/HD* low lesion volume and high disability, *HL/HD* high lesion volume and high disability. Results (*p* < 0.05, FDR corrected) are overlaid onto the F map (green) in the MNI152 standard brain. The red–yellow color indicates areas of higher functional connectivity (FC) in A. LL/LD than HL/LD; B. LL/HD than HL/HD; C. LL/LD than LL/HD; D. HL/LD than HL/HD; E. HL/LD than LL/HD. The color bars represent t values

**Fig. 5:**
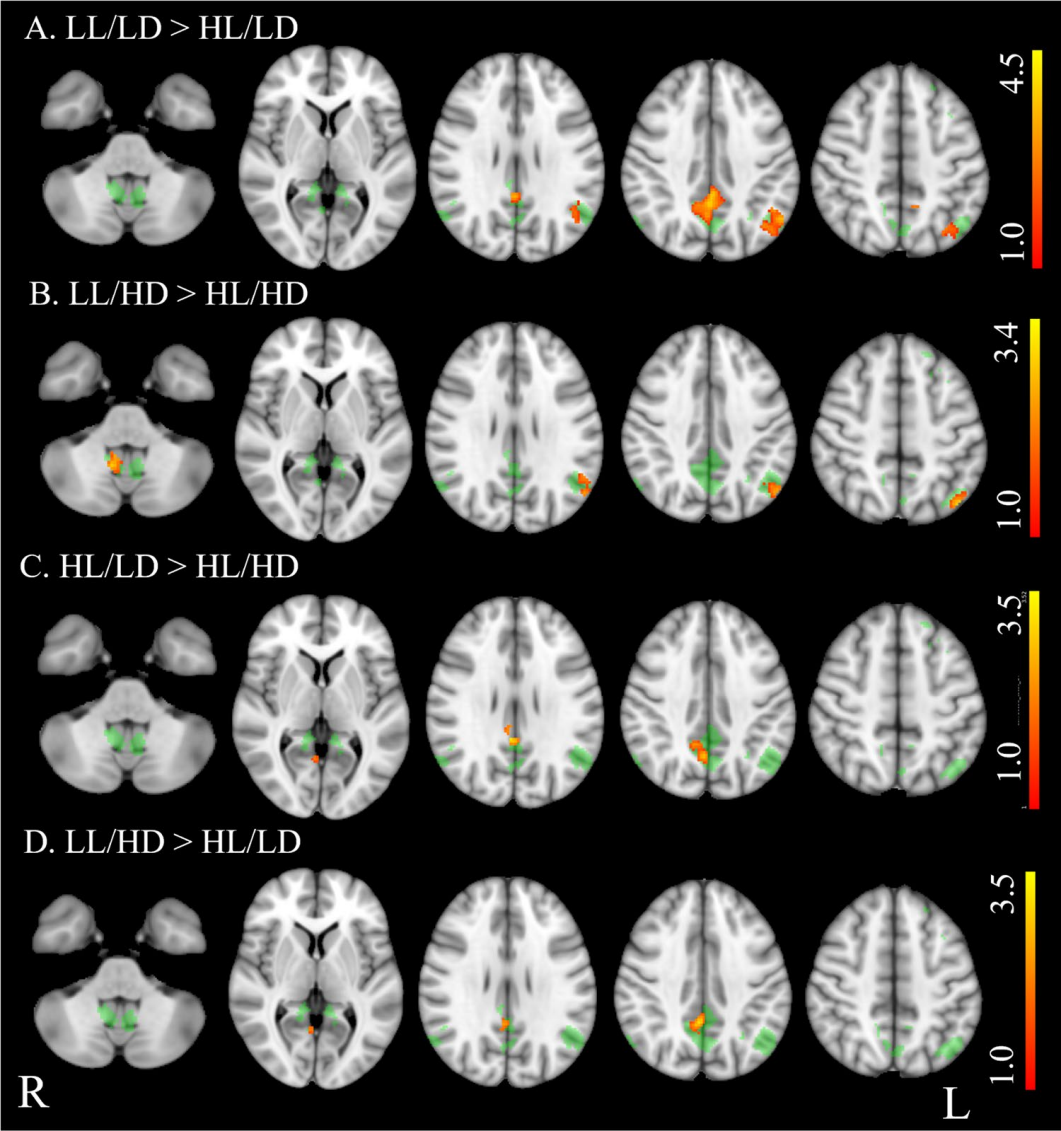
Group differences in resting-state functional connectivity among four groups of people with multiple sclerosis (PwMS) in the default mode network (DMN). *LL/LD* low lesion volume and low disability, *HL/LD* high lesion volume and low disability, *LL/HD* low lesion volume and high disability, *HL/HD* high lesion volume and high disability. Results (*p* < 0.05, FDR corrected) are overlaid onto the F map (green) in the MNI152 standard brain. The red–yellow color indicates areas of higher functional connectivity (FC) in A. LL/LD than HL/LD; B. LL/HD than HL/HD; C. HL/LD than HL/HD; D. LL/HD than HL/LD. The color bars represent t values

Table 1 shows demographics, clinical, and neuropsychological features in PwMS and differences from HS, while PwMS group differences are reported in Table 2. Brain structural MRI measures in PwMS and differences from HS are reported in Table 3, while PwMS group differences are shown in Fig. 1 and Table 4. Voxel-wise maps of the RSN FC difference between PwMS and HS are displayed in Figs. 2 and 3, and RSN FC differences among PwMS groups in Figs. 4 and 5. FC peak locations are all reported in Supplementary Tables 2, 3, 4 and 5.

**Table 1:** Demographic, clinical, and neuropsychological characteristics of people with multiple sclerosis and healthy controls

	HS *n* = 284	PwMS *n* = 717	LL/LD *n* = 242	HL/LD *n* = 138	LL/HD *n* = 121	HL/HD *n* = 216	*F*(*p*)
T2-hyperintense WM LV [ml]	–	8.0 (9.0)	2.1 (1.2)	10.9 (7.3)	2.5 (1.3)	15.7 (1.1)	189.9** (< 0.001)**
EDSSˆ	–	2.0 (0–9)	1.5 (0–2)	1.5 (0–2)	4.0 (2.5–8)	4.5 (2.5–9)	243.0 **(< 0.001)**
Females/males	186/98	513/204	184/58	104/34	86/35	139/77	12.1 **(0.06)**
Age [years]	41.9 (14.8)	40.9 (11.1)	35.6*** (9.6)	38.1 (9.4)	45.2 (10.7)	46.3** (10.5)	30.7 **(< 0.001)**
RRMS/PMS	–	557/48	239/0	138/0	74/23	106/25	87.8 **(< 0.001)**
Disease duration° [years]	–	11 (1–45)	5 (0–32)	9 (0–31)	11 (0–28)	17 (1–45)	72.2 **(< 0.001)**
9-HPT dominant hand [seconds]	19.0 (3.2)	24.6*** **(11.6)**	19.5 (3.0)	21.1 (3.9)	27.0*** **(16.2)**	31.9*** **(14.1)**	64.7 **(< 0.001)**
9-HPT non-dominant hand [seconds]	20.5 (4.0)	26.0*** **(10.7)**	20.7 (3.2)	22.7 (4.3)	26.9*** **(9.4)**	34.0*** **(14.8)**	82.6 **(< 0.001)**
25-FWT [seconds]	4.9 (1.6) (*n* = 160)	7.5*** (4.5) **(*n* = 525)**	6.0 (2.2) (*n* = 200)	6.2 (2.4) (*n* = 111)	9.7*** (5.8) **(*n* = 87)**	9.3*** **(6.1)** **(*n* = 127)**	40.1 **(< 0.001)**
PASAT 3 s [seconds]	41.9 (10.5) (*n* = 147)	37.8*** (13.4) **(*n* = 591)**	40.3 (13.1) (*n* = 207)	36.84 (12.1) (*n* = 118**)**	39.8 (13.0) (*n* = 100)	33.94*** (14.2) **(*n* = 166)**	9.89 **(< 0.001)**
SDMT	49.8 (11.9) (*n* = 82)	43.6*** (14.1) **(*n* = 472)**	50.5 (12.5) (*n* = 172)	43.8 (12.6) (*n* = 95)	44.5 (11.6) (*n* = 70)	33.93*** (13.0) **(*n* = 135)**	38.49 **(< 0.001)**

*HS* healthy subjects, *PwMS* people with Multiple Sclerosis, *LL/LD* low lesion volume and low disability, *HL/LD* high lesion volume and low disability, *LL/HD* low lesion volume and high disability, *HL/HD* high lesion volume and high disability, *n*. number of subjects, *RRMS* relapsing–remitting MS, *PMS* progressive MS, *EDSS* Expanded Disability status Scale, *LV* lesion volume, *9-HPT* nine-hole peg test, *25-FWT* timed 25 feet walking test, *PASAT* 3 s corrected paced auditory serial addition test with 3.0-s interstimulus interval, *SDMT* corrected symbol digit modalities test

Values are reported as average (standard deviation), if not stated otherwise. ˆEDSS is expressed as median (range). °Disease duration is expressed as mean (range). F and p values from the among groups ANCOVA are reported in the rightmost column of the table. In the other cells, bold values show significant difference from HS (Tukey test or Chi-square as relevant) after correction for multiple comparisons.

***p < 0.001

**p < 0.01

*p < 0.05

**Table 2:** Group differences of demographics, clinical, and neuropsychological data between healthy controls and the four groups of people with multiple sclerosis

	LL/LD vs HL/LD	LL/LD vs LL/HD	LL/LD vs HL/HD	HL/LD vs LL/HD	HL/LD vs HL/HD	LL/HD vs HL/HD
LV	** − 12.26, < 0.001**	** − **0.45, 1.000	** − 21.43, < 0.001**	**10.10, < 0.001**	** − 6.41, < 0.001**	** − 17.22, < 0.001**
Sex	0.00, 1.000	0.80, 1.000	6.94, 0.100	0.41, 1.000	4.24, 0.400	1.29, 1.000
Age [years]	−2.03, 1.000	** − 7.40, < 0.001**	** − 9.83, < 0.001**	** − 4.88, < 0.001**	** − 6.46, < 0.001**	** − **0.85, 1.000
RRMS/PMS	**–**	**57.17, < 0.001**	**45.93, < 0.001**	**33.64, < 0.001**	**26.81, < 0.001**	0.47, 4.946
Disease duration [years]	**– 3.88, 0.010**	** − 5.22, < 0.001**	** − 14.53, < 0.001**	** − **1.34, 1.000	** − 8.68, < 0.001**	** − 6.86, < 0.001**
9-HPT dominant hand [seconds]	** − **1.57, 5.161	** − 6.79, < 0.001**	** − 13.32, < 0.001**	** − 4.74, < 0.001**	** − 9.94, < 0.001**	** − 4.33, 0.002**
9-HPT-non-dominant hand [seconds]	** − **2.06, 2.36	** − 6.17, < 0.001**	** − 15.66, < 0.001,**	** − 3.77, 0.016**	** − 11.49, < 0.001**	** − 6.86, < 0.001,**
25-FWT [seconds]	** − **0.63, 1.000	** − 7.87, < 0.001**	** − 7.89, < 0.001**	**−6.53, < 0.001**	** − 6.31, < 0.001**	** − **0.83, 1.000
PASAT 3 s [seconds]	2.39,1.00	0.33,1.00	**4.84, < 0.001**	** − **1.72,1.00	1.89,1.00	**3.64,0.02**
SDMT	**4.21, 0.002**	3.40, 0.06	**11.62, < 0.001**	** − **0.34, 1.00	**5.95, < 0.001**	**5.75, < 0.001**

*LL/LD* low lesion volume and low disability, *HL/LD* high lesion volume and low disability, *LL/HD* low lesion volume and high disability, *HL/HD* high lesion volume and high disability, *RRMS* relapsing–remitting Multiple Sclerosis, *PMS* progressive Multiple Sclerosis, *EDSS* Expanded Disability status Scale, *LV* lesion volume, 9-*HPT* nine-hole peg test, *25-FWT* timed 25 feet walking test, *PASAT* 3 s paced auditory serial addition test with 3.0-s interstimulus interval, *SDMT* symbol digit modalities test

Differences between groups are reported as *t* value and *p* value. Bold values show significant differences (Tukey test) after correction for multiple comparisons

**Table 3:** Structural brain volumes of people with multiple sclerosis, divided into four groups, and healthy controls

	HS	LL/LD	HL/LD	LL/HD	HL/HD	*F*(*p*)
Global brain volumes [%]						
Gray matter	40.45 ± 4.35	40.95 ± 4.12	39.11 ± 4.07***	**39.46 ± 4.05**	37.23 ± 4.33***	31.7 (< 0.001)
White matter	40.9 ± 3.81	41.51 ± 3.82	40.19 ± 3.89	40.17 ± 3.99	38.69 ± 4.49***	15.9 (< 0.001)
TIV [cm^3^]	1432.31 ± 147.7	1412.42 ± 130.96	1406.59 ± 143.18	1389.95 ± 142.5	1341.9 ± 93.79***	19.1 (< 0.001)
Subcortical volumes [%]						
Thalamus	1.02 ± 0.07	1.01 ± 0.08*	0.91 ± 0.11***	0.98 ± 0.08***	0.85 ± 0.11***	171.9 (< 0.001)
Caudate	0.48 ± 0.06	0.48 ± 0.06	0.43 ± 0.05***	0.46 ± 0.06	0.40 ± 0.05***	95.5 (< 0.001)
Putamen	0.64 ± 0.07	0.63 ± 0.06	0.57 ± 0.08***	0.61 ± 0.07	0.53 ± 0.09***	92.9 (< 0.001)
Pallidum	0.23 ± 0.03	0.22 ± 0.03	0.21 ± 0.03***	0.23 ± 0.03	0.20 ± 0.04***	32.9 (< 0.001)
Cerebellar volume [%]						
Cerebellum	8.35 ± 0.63	8.41 ± 0.58	8.28 ± 0.61	8.11 ± 0.78*	7.78 ± 0.81***	36.58 (< 0.001)
Spinal cord area [cm^2^]						
C2–C3 area	0.65 ± 0.07	0.63 ± 0.09	0.61 ± 0.08	0.57 ± 0.09***	0.55 ± 0.09***	40.9 (< 0.001)

*HC* healthy controls, *LL/LD* low lesion volume and low disability, *HL/LD* high lesion volume and low disability, *LL/HD* low lesion volume and high disability, *HL/HD* high lesion volume and high disability

Brain structure volumes are reported as a percentage of the total intracranial volume (TIV). Subcortical volumes are considered as the sum of the right and left values are displayed as mean ± standard deviation. *F* and *p* values from the among groups ANCOVA are reported in the rightmost column of the table. In the other cells, bold values show significant difference from HC (Tukey test) after correction for multiple comparisons

^***^(*p* < 0.001)

^**^(*p* < 0.01)

^*^(*p* < 0.05)

**Table 4:** Group differences of structural magnetic resonance imaging measures between healthy controls and the four groups of people with multiple sclerosis

	LL/LD vs HL/LD	LL/LD vs LL/HD	LL/LD vs HL/HD	HL/LD vs LL/HD	HL/LD vs HL/HD	LL/HD vs HL/HD
Global brain volumes						
GM	**3.76, 0.017**	0.85, 1.000	**6.16, < 0.001**	− 2.4, 1.000	1.83, 1.000	**4.45, 0.001**
WM	2.96, 0.255	2.5, 1.000	**6.8, < 0.001**	0.23, 1.000	3.15, 0.145	3.34, 0.079
TIV	0.48, 1.00	2.12, 1.00	**5.02, < 0.001**	1.51, 1.00	**4.06, 0.01**	2.38, 1.00
Deep gray volumes						
Thalamus	**10.59, < 0.001**	2.12, 1.000	**17.61, < 0.001**	− **7.02, < 0.001**	**5.37, < 0.001**	**13.04, < 0.001**
Caudate	**8.52, < 0.001**	1.17, 1.000	**12.82, < 0.001**	− **6.13, < 0.001**	3.14, 0.146	**9.87, < 0.001**
Putamen	**7.59, < 0.001**	0.88, 1.000	**13.2, < 0.001**	− **5.61, < 0.001**	**4.36, 0.002**	**10.49, < 0.001**
Pallidum	**5.37, < 0.001**	− 0.66, 1.000	**9.44, < 0.001**	− **3.94,0.009**	3.17, 0.132	**7.46, < 0.001**
Cerebellar volumes						
Cerebellum	1.56, 1.000	2.89, 0.317	**8.00, < 0.001**	1.31, 1.000	**5.56, < 0.001**	**3.99, 0.007**
Spinal cord area						
C2–C3 area	0.84, 1.000	**4.44, 0.001**	**7.67, < 0.001**	3.3, 0.088	**6.07, < 0.001**	2.34, 1.000

*LL/LD* low lesion volume and low disability, *HL/LD* high lesion volume and low disability, *LL/HD* low lesion volume and high disability, *HL/HD* high lesion volume and high disability. Differences between groups are reported as *t* value and *p* value. Bold values show significant differences (Tukey test) after correction for multiple comparisons.

### Group-effect analysis

ANCOVA revealed significant group effects for all demographic, clinical and neuropsychological features, except for sex (Table 1). Additionally, ANCOVA showed group effects for all structural MRI measures, including volumes of WM, GM, thalamus, caudate, putamen, pallidum, cerebellum, and spinal cord area (see Table 3). A significant group effect for FC was found in several regions of the SMN, including the insula, precentral and post-central gyri, and the supplementary motor area (SMA), and of the DMN, specifically in the left superior frontal gyrus, bilateral posterior cingulate cortex, precuneus, lateral parietal cortex and cerebellum (F maps are shown in Figs. 2, 3, 4 and 5). No group effect was found in the other 4 RSNs.

### PwMS vs healthy subjects

The LL/LD group was younger than HS, while the HL/HD group was older. At the motor function tests, i.e., 9-HPT and 25-FWT, PwMS with high disability (LL/HD and HL/HD) obtained significantly higher scores than HS, while PwMS with low disability (LL/LD and HL/LD) performed similarly to HS. Difference in average PASAT and SDMT scores shows that HL/HD alone scored significantly lower than HS (Tables 1). However, when looking at the frequency, a large percentage of patients with high lesion load and low disability performed worse than HS at least at one cognitive test (Supplementary Table 6).

Table 3 shows the values of all MRI measures in both HS and PwMS. Compared with HS, LL/LD had reduced thalamic volume alone, while LL/HD had reduced volumes of GM, thalamus, cerebellum, and SC area. HL/LD had reduced volumes in all structures except WM, cerebellum, and SC area, while HL/HD had reduced volumes of all brain structures and SC area.

Compared with HS, LL/LD showed increased RS-FC within most areas of the SMN, including the insula, pre- and post-central gyri, and SMA (Fig. 2A). Conversely, all the other groups of PwMS had reduced RS FC: HL/LD within the right insula (Fig. 2B); LL/HD within the right post-central gyrus (Fig. 2C); HL/HD within the insula, pre- and post-central gyri, and SMA (Fig. 2D). Compared to HS, all groups of PwMS showed a reduced RS-FC of the DMN that involved the precuneus alone in LL/LD (Fig. 3A), precuneus, posterior cingulate gyrus, left parietal cortex in HL/LD (Fig. 3B) and LL/HD (Fig. 3C), and precuneus, posterior cingulate gyrus, parietal cortex bilaterally, cerebellum, and left superior frontal gyrus in HL/HD (Fig. 3D).

### The dissociation groups

To focus on the aim of this work here, we describe only results for the two dissociation groups (HL/LD and LL/HD), while results of comparisons of clinical, neuropsychological, and MRI data of all patient groups are shown in Tables 2 and 4, and described in supplementary material.

### Dissociation 1: PwMS with high lesion volume and low disability (HL/LD)

About 20% of cases showed low physical disability despite a high LV, and 17% and 16% of them scored less than the cutoff at at least one cognitive test (PASAT3 and SDMT, respectively, see Supplementary Table 6). They showed worse cognitive performance than HS, while upper and lower limb abilities were preserved. They also showed longer disease duration and lower SDMT scores than LL/LD (Tables 1 and 2).

Concerning structural MRI variables, HL/LD had lower volumes of GM, thalamus, caudate, putamen, and pallidum than HS and LL/LD, while WM, cerebellum, and spinal cord showed comparable values. Compared to HL/HD, they showed higher thalamus, putamen, and cerebellum volumes and SC area (Fig. 1, Tables 3 and 4).

HL/LD had lower SMN-RS-FC in a small area corresponding to the right insula than HS (Fig. 2B) and lower SMN-RS FC in the insula, pre- and post-central cortex, and SMA, bilaterally than LL/LD (Fig. 4A). Compared to HL/HD, they had higher SMN-RS-FC in the bilateral pre- and post-central cortex and SMA (Fig. 4D).

HL/LD had lower DMN RS-FC in the posterior cingulum/precuneus and in the left lateral parietal cortex than HS (and 3B) and LL/LD (Fig. 5A). Compared to HL/HD, they had higher DMN FC in the right posterior cingulum/precuneus (Fig. 5C).

### Dissociation 2: PwMS with low lesion volume and high disability (LL/HD)

PwMS with high disability despite low LV were about 17% of our sample. They performed worse in all motor tests than HS (Table 1) and 11% of them scored less than the cutoff at at least one cognitive test (see Supplementary Table 6). They were older, with a longer disease duration and included a higher number of progressive phenotypes than LL/LD. They performed better than HL/HD at all motor and cognitive tests, except for the 25-FWT (Table 2).

They showed lower volumes of GM, thalamus, cerebellum, and SC area than HS (Table 3). Only SC area was reduced in LL/HD with respect to LL/LD, while all MRI variables, except for the WM volume and SC area, were less severely affected in LL/HD than in HL/HD (Fig. 1, Table 4).

LL/HD had lower SMN-RS-FC in a small area corresponding to the right precentral cortex than HS (Fig. 2C) and lower SMN-RS-FC in the insula, pre- and post-central cortex and SMA, bilaterally than LL/LD (Fig. 4C). Compared to HL/HD, they had higher SMN-RS FC in left insula, right pre–post-central cortex, and SMA (Fig. 4B).

LL/HD had lower DMN RS-FC in the posterior cingulum/precuneus and in the left lateral parietal cortex than HS (Fig. 3C), a similar pattern of DMN RS-FC as LL/LD, while they had higher DMN RS FC in the right cerebellum and left lateral parietal cortex than HL/HD (Fig. 5B).

## Discussion

Using an objective method to define the cutoff of LV and disability based on the respective medians, we found that the clinico-radiological dissociation was present in more than one-third of cases of a large dataset, suggesting that this phenomenon is non-negligible in clinical practice. This percentage is higher than that reported by Healy et al. [29], who described a total of 13.5% PwMS with clinical/MRI dissociation by dividing the sample using a more arbitrary cutoff to select the extreme cases of highest or lowest LV and disability. On the other hand, a better association between clinical and radiological measures and, presumably, a lower percentage of PwMS presenting the clinico-radiological dissociation could be found if more complete and specific tools were used for both clinical and radiological evaluation [8–11, 14, 30].

However, in the diagnostic workup and in clinical practice, clinical scales assessing disability in all domains of human activities and software-processed MRI data are not generally used. Our results indicate that some efforts must be made to better evaluate the patient’s clinical status and/or overall CNS damage, especially when WM lesion burden and EDSS are not congruent.

### High lesion volume associated with low disability

Our results indicate that EDSS alone cannot describe the real grade of severity of the clinical picture, mainly due to underestimation of cognitive abilities [4, 31]. Cognitive dysfunction was present in PwMS despite low EDSS scores (< 2), underlying that cognitive impairment can occur even in PwMS showing an apparent good clinical status [32].

Similarly, evaluation of thalamus and basal ganglia volumes would lead to a better definition of CNS damage. In HL/LD, a high volume of WM lesions was associated with atrophy of GM, thalamus, and basal ganglia. Atrophy of thalamus and basal ganglia are not always associated, since thalamic volume loss was present in all groups, while basal ganglia tissue loss only in PwMS with high LV. Loss of thalamic volume is one of the earliest and most evident findings of GM pathology in MS [33], and has been strongly associated with various neurological symptoms, especially cognitive impairment [34]. Basal ganglia atrophy, on the other hand, is likely driven by WM lesions [35] and data on the association between basal ganglia atrophy and physical disability are limited and inconclusive [36]. As well, the evaluation of SC atrophy may aid the definition of the CNS damage, since it is associated with upper and lower limb impairment regardless of the lesion volume.

In this dissociation group, upper and lower limb abilities were preserved, likely due to sparing key structures for motor abilities, such as the cerebellum and spinal cord [37, 38].

Defining the role of RS-FC may be more complex due to difficulties attributing the meaning of adaptive or maladaptive plasticity to connectivity changes in cross-sectional studies [39, 40]. Increased RS-FC in the SMN with respect to HS, involving almost the entire network, was observed only in LL/LD. This finding supports the hypothesis that RS-FC cannot increase if a certain degree of atrophy is already developed [41]. With respect to HS, HL/LD showed a substantially maintained activity at rest in the SMN except for a circumscribed spot in the left insula. This finding partially contrasts with the previous studies indicating that preserved upper limb function is associated with higher FC in motor and non-motor regions [39, 42, 43]. On the other hand, the DMN, whose function is related to several high-order functions [44], showed decreased FC in HL/LD with respect to HS, paralleling low scores obtained at neuropsychological tests, in agreement with the previous studies [45].

In conclusion, our results add evidence to the importance of the cognitive dysfunction and volumetric changes occurring in MS, especially when low EDSS scores are associated with high volume of WM lesions. Therefore, the clinico-radiological paradox may be partially explained by the specificity of the test used to assess clinical status, being the EDSS biased toward mobility.

### Low lesion volume associated with high disability

Our results indicate that WM LV alone cannot describe the real grade of severity of overall CNS damage and explain the high EDSS in LL/HD, mainly due to underestimation of brain and spinal cord atrophy. PwMS with high disability despite the low WM lesion volume showed impairment of both motor abilities and cognitive functions associated with atrophy of GM, thalamus, cerebellum, and spinal cord.

Spinal cord atrophy can explain the reduced upper and lower limb abilities, while thalamic and cerebellar atrophy was likely responsible for impaired upper limb and cognitive dysfunction.

Healy et al. [29] described more cervical but not thoracic lesions in LL/HD than in HL/LD, but they did not report cervical cord atrophy measures. On the other hand, the leading role of spinal cord atrophy in determining motor disability is well-known [46]. In our study atrophy of the spinal cord was always associated with a high disability, regardless of the severity of the brain lesion burden, in keeping with the previous studies [37, 47].

Similarly, the association between thalamic integrity and cognitive abilities, and that between cerebellar integrity and both motor coordination and cognitive performance is unquestioned [38, 48]. Cerebellar volume loss in MS has been described in several studies [49, 50]. While correlations of cerebellar volume with EDSS were found to be moderate [47] or absent [50], a more robust correlation was found between cerebellar atrophy and fine motor skills [49] or cognitive dysfunction [51].

In conclusion, a routine evaluation of the spinal cord would be of primary importance in the clinical assessment of PwMS with low lesion volume, especially if associated with a high EDSS. Likewise, the evaluation of cerebellar and thalamic volumes would be useful for disability monitoring [38, 48].

### Strengths and limitations

A strength of this study is the large sample of PwMS collected with broad inclusion criteria by four MS centers, which reflects the distribution of PwMS in the real world and makes results highly reliable. Another strength is the multimodal aspect of the study, which included many structural and functional MRI measures and motor/neuropsychological scores, providing a series of information useful not only for clinical patient management but also for consideration of pathophysiological mechanisms of the disease.

On the other hand, this study has some limitations.

Cognitive tests were not available for the entire sample; they were, however, obtained in a high percentage of PwMS. In this study, we did not consider a complete battery of neuropsychological tests. However, we used PASAT3 and SDMT, which are the most used tests to assess disturbances in attention, information processing speed, memory, and executive skills in MS [52, 53].

To devise PwMS into four groups by means an objective method, we used EDSS and LV medians. This led to a skew toward low values of EDSS, LV and disease duration, making these results not generalizable to the entire MS population. On the other hand, to devise PwMS according to other methods, e.g., clinical cutoff of EDSS = 4 (https://www.mssociety.org.uk/living-with-ms/treatments-and-therapies/getting-treatment-for-ms/expanded-disability-status-scale), was not feasible in this series, since it would have led to numerically unbalanced groups and statistical inaccuracy.

MRI was acquired at different scanners, causing center-specific effects on the data. However, this bias was removed using harmonization tools [28].

Further, although several MRI parameters have been included in this analysis, some other measures associated with disability, e.g., cortical lesions [54] or white matter integrity loss [55], were not. We excluded cortical lesion assessment, since sequences able to image cortical lesions at 3 T, e.g., DIR or PSIR, were available in a low percentage of patients alone. We also excluded DTI data from the analysis, because, although obtained in all patients, acquisitions with different scanners and sequences may introduce systematic errors in DTI parameter estimation [56].

Finally, spinal cord atrophy was assessed by measuring the cervical cord area at the C2–C3 level alone, which has been found to be a reliable measure [37].

## Conclusions

Our study on a large population using multiple clinical and radiological measures indicates that the clinico-radiological dissociation may be found in a relevant number of PwMS, at least in this large cohort recruited from four Italian MS centers, making this issue noteworthy in daily clinical practice.

Our results indicate that when WM lesion burden and EDSS are not congruent, some aspects of the clinical picture or of the CNS damage evaluated by MRI must be more carefully considered. When the EDSS score is low despite high lesion volume, a more complete clinical evaluation, especially the assessment of the cognitive profile, is necessary and can lead to a better definition of the real patient's clinical status. The hypothesized role of FC as a compensatory mechanism of neuroplasticity to maintain neurological functions despite brain damage deserves some consideration. Assuming that the FC increase of the SMN is beneficial and aimed at maintaining motor function despite brain damage [40], our findings indicate that FC increase is possible only if brain structural integrity is preserved. Therefore, the role of FC in explaining the incongruity between low physical disability and high lesion burden should be reconsidered.

On the other hand, when the EDSS score is higher than expected due to the number of brain WM lesions, the spinal cord evaluation becomes mandatory [57]. This study also underlines the role of some key CNS structures, besides the spinal cord, in disability accumulation, such as the thalamus and cerebellum. Thalamic volume loss is a frequent finding observed in all four groups, even if it is associated with high lesion volume to a greater extent. Cerebellar volume loss, on the other hand, likewise spinal cord involvement, is a marker of physical disability, since it was found only in the two groups showing high EDSS scores. Thus, preserving the integrity of the cerebellum and spinal cord may be the key to maintaining low levels of physical disability. Recently, especially thanks to the development of AI-based algorithms, it has been improved the access to tools able to easily estimate anatomical measures on MR images. Therefore, it is not impossible to expect, in the next future, the possibility for radiologists to access anatomical structures’ volumetry while analyzing the clinical MRI of PwMS. This study strongly supports the development of clinical technology toward this endpoint, to have clinicians able to periodically evaluate thalamic and cerebellar volumes and SC area, together with PASAT and/or SDMT, during the routinary clinical practice to best quantify disability and MS pathology load.

## Conflict of interest

AO, ST, CP, VB, EG, ADA, MA, and NT: nothing to disclose. AG: has received speaker and consulting fees from Biogen, Genzyme, Merck Serono, Mylan, Novartis, Roche, and Teva, and receives research support from Fondazione Italiana Sclerosi Multipla. NDS: has received honoraria from Biogen-Idec, Bristol Myers Squibb, Celgene, Genzyme, Immunic, Merck Serono, Novartis, Roche, and Teva for consulting services, speaking, and travel support. He serves on advisory boards for Merck, Novartis, Biogen-Idec, Roche, and Genzyme, Immunic, and he has received research grant support from the Italian MS Society. RC: was awarded an MAGNIMS-ECTRIMS fellowship in 2019; she received speaker honoraria from Roche, Merck Serono, and Sanofi, and travel support for conferences by Novartis. PV: has received speaker honoraria from Biogen Idec. CP: has served on scientific advisory boards for Novartis, Merck, Biogen, Sanofi, Genzyme, Teva, and Actelion; received funding for travel and speaker honoraria from Biogen, Teva, Sanofi Genzyme, Actelion, and Novartis; received research support from Biogen, Teva, Novartis, and Genzyme. MAR: has received consulting fees from Biogen, Bristol Myers Squibb, Eli Lilly, Janssen, Roche; and speaker honoraria from AstraZaneca, Biogen, Bristol Myers Squibb, Bromatech, Celgene, Genzyme, Horizon Therapeutics Italy, Merck Serono SpA, Novartis, Roche, Sanofi and Teva. She receives research support from the MS Society of Canada, the Italian Ministry of Health, the Italian Ministry of University and Research, and Fondazione Italiana Sclerosi Multipla. She is Associate Editor for Multiple Sclerosis and Related Disorders. MF: is the Editor-in-Chief of the Journal of Neurology, Associate Editor of Human Brain Mapping, Neurological Sciences, and Radiology; received compensation for consulting services from Alexion, Almirall, Biogen, Merck, Novartis, Roche, Sanofi; speaking activities from Bayer, Biogen, Celgene, Chiesi Italia SpA, Eli Lilly, Genzyme, Janssen, Merck-Serono, Neopharmed Gentili, Novartis, Novo Nordisk, Roche, Sanofi, Takeda, and TEVA; participation in Advisory Boards for Alexion, Biogen, Bristol-Myers Squibb, Merck, Novartis, Roche, Sanofi, Sanofi-Aventis, Sanofi-Genzyme, Takeda; scientific direction of educational events for Biogen, Merck, Roche, Celgene, Bristol-Myers Squibb, Lilly, Novartis, Sanofi-Genzyme; he receives research support from Biogen Idec, Merck-Serono, Novartis, Roche, the Italian Ministry of Health, the Italian Ministry of University and Research, and Fondazione Italiana Sclerosi Multipla. PP: has received funding for travel from Novartis, Genzyme, and Bracco and speaker honoraria from Biogen.

## Supplementary Information

Below is the link to the electronic supplementary material.

Supplementary file1 (DOCX 37 KB)

## Data Availability

The data that support the findings of this study are available on request from the corresponding author. The data are not publicly available due to privacy or ethical restrictions.
