# Supplementary material for: Clinical and MRI features contributing to the clinico-radiological dissociation in a large cohort of people with multiple sclerosis
Source: J Neurol. 2025 Apr 9;272(5):327. doi: 10.1007/s00415-025-12977-6 (PMC11982092; doi:10.1007/s00415-025-12977-6)
Supplement: Supplementary file 1 — Supplementary file1 (DOCX 37 KB) [file 415_2025_12977_MOESM1_ESM.docx]

**Supplementary Materials.**

**Title:** **Clinical and MRI features contributing to the clinico-radiological dissociation in a large cohort of people with Multiple Sclerosis.**

**MRI analysis**

*Data preprocessing*

Preprocessing of functional and structural images was performed using fMRIPrep 20.2.3, a toolbox based on Nipype 1.5.0 (RRID: SCR_002502) [1, 2]. Each of the T1-weighted (T1w) images were preprocessed with the following pipeline: first, the T1w image was corrected for intensity non-uniformity (INU) with N4BiasFieldCorrection [3], distributed with ANTs 2.3.3 ( [4] , RRID:SCR_004757), and used as T1w-reference throughout the workflow. The T1w-reference was then skull-stripped with a Nipype implementation of the antsBrainExtraction.sh workflow (from ANTs), using OASIS30ANTs as target template. Brain tissue segmentation of cerebrospinal fluid (CSF), white-matter (WM) and gray-matter (GM) was performed on the brain-extracted T1w using fast (FSL 5.0.9, RRID:SCR_002823, [5] ). Brain surfaces were reconstructed using recon-all (FreeSurfer 6.0.1, RRID:SCR_001847, [6] ), and the brain mask estimated previously was refined with a custom variation of the method to reconcile ANTs-derived and FreeSurfer-derived segmentations of the cortical gray-matter of Mindboggle (RRID:SCR_002438, [7]). Volume-based spatial normalization to two standard spaces (MNI152NLin2009cAsym, MNI152NLin6Asym) was performed through nonlinear registration with antsRegistration (ANTs 2.3.3), using brain-extracted versions of both T1w reference and the T1w template.

The following templates were selected for spatial normalization: ICBM 152 Nonlinear Asymmetrical template version 2009c ( [8] , RRID:SCR_008796; TemplateFlow ID: MNI152NLin2009cAsym), FSL’s MNI ICBM 152 non-linear 6th Generation Asymmetric Average Brain Stereotaxic Registration Model ( [9], RRID:SCR_002823; TemplateFlow ID: MNI152NLin6Asym].

Functional preprocessing was also performed using fMRIPrep. For each subject, the following preprocessing was performed: First, a reference volume and its skull-stripped version were generated using a custom methodology of fMRIPrep. The BOLD reference was then co-registered to the T1w reference using bbregister (FreeSurfer) which implements boundary-based registration [10]. Co-registration was configured with six degrees of freedom. Head-motion parameters with respect to the BOLD reference (transformation matrices, and six corresponding rotation and translation parameters) are estimated before any spatiotemporal filtering using mcflirt (FSL 5.0.9, [11] ). BOLD runs were slice-time corrected using 3dTshift from AFNI 20160207 ( [12] , RRID:SCR_005927). The BOLD time-series (including slice-timing correction when applied) were resampled onto their original, native space by applying the transforms to correct for head-motion. These resampled BOLD time-series will be referred to as preprocessed BOLD in original space, or just preprocessed BOLD. The BOLD time-series were resampled into standard space, generating a preprocessed BOLD run in MNI152NLin2009cAsym space. First, a reference volume and its skull-stripped version were generated using a custom methodology of fMRIPrep. Automatic removal of motion artifacts using independent component analysis [13] was performed on the preprocessed BOLD on MNI space time-

series after removal of non-steady state volumes and spatial smoothing with an isotropic, Gaussian kernel of 6mm FWHM (full-width half-maximum). Corresponding “non-aggressively” denoised runs were produced after such smoothing. Additionally, the “aggressive” noise-regressors were collected and placed in the corresponding confounds file. Functional preprocessed data were finally subjected to WM and CSF signal regression and high-pass filtering (100-seconds cut-off) [13].

**Results**

**PwMS with low disability and different lesion volume (LL/LD and HL/LD)**

Demographic/clinical measures in PwMS with low disability but different lesion volumes differed in disease duration and SDMT scores, respectively longer and lower in the HL/LD group (Table 1, Table 2, Supplementary Table 6). Comparison of structural MRI variables between these two groups showed differences in GM, thalamus, putamen, pallidum, and caudate volumes, which were more affected in HL/LD (Figure 1, Table 3 and 4). The LL/LD had higher SMN resting state functional connectivity (RS-FC) than HL/LD within most SMN, including bilateral insula, pre- and post-central gyri, and supplementary motor area (Figure 4A, Supplementary Table 4). Likewise, RS-FC was higher in LL/LD than HL/LD in the DMN, involving in the precuneus and left lateral parietal cortex (Figure 5A, Supplementary Table 5).

**PwMS with high disability and different lesion volume (LL/HD and HL/HD)**

In PwMS with a high disability but different lesion volume, disease duration, and neuropsychological scores were significantly different, except for 25-FWT, with HL/HD being more affected. Except for the SC area, a comparison of structural MRI variables between these two groups showed differences in all the structures, which were more severely affected in HL/HD (Figure 1, Table 3 and 4). LL/HD had higher SMN RS-FC than HL/HD in the left insula, right pre- and postcentral gyri, and supplementary motor area (Figure 4B, Supplementary Table 4). RS-FC was higher in LL/HD than HL/HD in some areas of the DMN, including the cerebellum and left lateral parietal cortex (Figure 5B, Supplementary Table 5).

**PwMS with low lesion volume and different disabilities (LL/LD and LL/HD)**

Demographic/clinical measures in PwMS with low lesion volume but different disabilities were all significantly different, except for sex, SDMT and PASAT, being LL/HD older and more affected (Table 2). Comparison of structural MRI variables between these two groups showed significance in the SC area alone, which was smaller in LL/HD (Figure 1, Table 3 and 4). The LL/LD had higher RS-FC than LL/HD within most SMN, including bilateral insula and pre- and postcentral gyri and supplementary motor cortex (Figure 4C, Supplementary Table 4). No differences in DMN RS-FC were found between these two groups.

**PwMS with high lesion volume and different disabilities (HL/LD and HL/HD)**

Demographic/clinical measures in PwMS with high lesion volume but different disabilities were all significantly different except for sex and PASAT, with the highly disabled PwMS being more affected (Table 2). Comparison of structural MRI variables between these two groups showed significant differences in most of the structural measures, i.e., cerebellum, thalamus and putamen volumes, and spinal cord area, with patients with higher disability showing greater atrophy of the structures mentioned above (Figure 1, Table 2 and 4). The HL/LD had higher SMN RS-FC than HL/HD within the bilateral precentral gyrus and supplementary motor cortex (Figure 4D, Supplementary Table 4). The HL/LD also had higher DMN RS-FC than HL/HD within the right posterior cingulate cortex (Figure 5C, Supplementary Table 5).

Since HL/HD also showed T2 lesion volume significantly higher than HL/LD, to avoid any dependencies of the results from the focal lesions’ amount, we repeated the analysis after removing from HL/HD those subjects with the highest LV so to make the two groups comparable (data are shown in S Table 7). This sub-analysis confirmed that sensorimotor and cognitive cerebellum, thalamus, putamen and spinal cord were significantly more affected in HL/HD than in HL/LD (t=6.89 p<0.001, t=4.01 p<0.001, t=7.00 p<0.03, t=1.54 p<0.02 and t=4.79 p<0.001, respectively), while GM, WM, pallidum and caudate volumes remained comparable.

**PwMS in the Clinical/MRI mismatch groups (LL/HD and HL/LD)**

The clinical/MRI mismatch groups differed in age and the performance of the motor function tests, being LL/HD older and slower than HL/LD. On the contrary, in the cognitive tests, they performed comparably (Table 1 and 2). LL/HD had GM, deep GM, and cerebellum volumes larger than HL/LD (Figure 1, Table 3 and 4). HL/LD had higher SMN RS-FC than LL/HD within the bilateral precentral gyrus (Figure 4E, Supplementary Table 4) and lower DMN RS-FC in the right posterior cingulate cortex (Figure 5D, Supplementary Table 5).

**References**

1. Esteban, O., Markiewicz, C.J., Blair, R.W. et al. (2019). fMRIPrep: a robust preprocessing pipeline for functional MRI. Nat Methods 16, 111–116. <https://doi.org/10.1038/s41592-018-0235-4>
2. Gorgolewski, K., Burns, C.D., Madison, C.et al. (2011). Nipype: a flexible, lightweight and extensible neuroimaging data processing framework in python. Front Neuroinform 5, 13. <https://doi.org/10.3389/fninf.2011.00013>
3. Tustison NJ, Avants BB, Cook PA, et al (2010) N4ITK: Improved N3 Bias Correction. IEEE

Transactions on Medical Imaging 29:1310–1320. <https://doi.org/10.1109/TMI.2010.2046908>

1. Avants BB, Epstein CL, Grossman M, Gee JC (2008) Symmetric diffeomorphic image registration with cross-correlation: Evaluating automated labeling of elderly and neurodegenerative brain. Medical Image Analysis 12:26–41. https://doi.org/10.1016/j.media.2007.06.004
2. Zhang Y, Brady M, Smith S (2001) Segmentation of brain MR images through a hidden Markov random field model and the expectation-maximization algorithm. IEEE Transactions on Medical Imaging 20:45–57. https://doi.org/10.1109/42.906424
3. Dale AM, Fischl B, Sereno MI (1999) Cortical Surface-Based Analysis: I. Segmentation and

Surface Reconstruction. NeuroImage 9:179–194. https://doi.org/10.1006/nimg.1998.0395

1. Klein A (2017) Mindboggling morphometry of human brains. https://doi.org/10.1371/journal.pcbi.1005350
2. Fonov V, Evans A, McKinstry R, et al (2009) Unbiased nonlinear average age-appropriate brain templates from birth to adulthood. NeuroImage 47:S102. https://doi.org/10.1016/S1053-8119(09)70884-5
3. Evans AC, Janke AL, Collins DL, Baillet S (2012) Brain templates and atlases. NeuroImage

62:911–922. https://doi.org/10.1016/j.neuroimage.2012.01.024

1. Greve DN, Fischl B (2009) Accurate and robust brain image alignment using boundary-based

registration. Neuroimage 48:63–72. https://doi.org/10.1016/j.neuroimage.2009.06.060

1. Jenkinson M, Bannister P, Brady M, Smith S (2002) Improved optimization for the robust and accurate linear registration and motion correction of brain images. Neuroimage 17:825–841
2. Cox RW, Hyde JS (1997) Software tools for analysis and visualization of fMRI data. NMR

Biomed 10:171–178. https://doi.org/10.1002/(sici)1099-1492(199706/08)10:4/5&lt;171::aid-

nbm453&gt;3.0.co;2-l

1. Pruim RHR, Mennes M, van Rooij D, et al (2015) ICA-AROMA: A robust ICA-based strategy for removing motion artifacts from fMRI data. Neuroimage 112:267–277. <https://doi.org/10.1016/j.neuroimage.2015.02.064>

**Supplementary Table 1: MRI acquisition parameters for each center participating in INNI.** INNI: Italian Neuroimaging Network Initiative; FOV: field of view; TR: repetition time; TE: echo time; TI: inversion time; FA: flip angle; ETL: echo train length; TA: acquisition time; TFE: turbo field echo; IR: inversion recovery; FSPGR: fast spoiled gradient echo; MPRAGE: magnetization prepared gradient echo; FFE fast field echo.

|  | **Center A** | | | **Center B** | | | **Center C** | | | **Center D** | | |
| --- | --- | --- | --- | --- | --- | --- | --- | --- | --- | --- | --- | --- |
| MR scanner | Philips medical system—intera | | | GE medical system signa HDxt | | | Siemens Verio | | | Philips medical system—achieva | | |
| MR imaging sequence | Dual-echo | T_1_-weighted TFE | T2*-weighted single-shot EPI | T_2_-weighted FLAIR | T_1_-weighted IR–FSPGR | T2*-weighted gradient-echo EPI | Dual-echo | T_1_-weighted MPRAGE | T2*-weighted single-shot EPI | Dual-echo | T_1_-weighted FFE | T2*-weighted EPI |
| Coil | 8-channel head coil | | | 8-channel head coil | | | 12-channel head coil | | | 32-channel head coil | | |
| Imaging plane | Axial | Axial | Axial | Axial | Sagittal | Axial | Axial | Sagittal | Axial | Axial | Axial | Axial |
| Acquisition voxel [mm^3^] | 1 × 1 × 3 | 1 × 1 × 1 | 1.87 x 1.87 x 4 | 1 × 1 × 3 | 1 × 1 × 1.2 | 4 x 4 x 4 | 1 × 1 × 4 | 1 × 1 × 1 | 3 x 3 x 3 | 1 × 1 × 3 | 1 ×1 × 1 | 1.87 x 1.87 x 4 |
| FOV [mm^2^] | 243 × 243 | 230 × 230 | 240 x 240 | 256 × 256 | 256 × 256 | 256 × 256 | 220 × 220 | 256 × 256 | 192 x 192 | 240 × 240 | 256 × 256 | 234 x 234 |
| TR [ms] | 2910 | 25 | 3000 | 9002 | 6.988 | 1508 | 5310 | 1900 | 3000 | 4000 | 10 | 3000 |
| TE [ms] | 16–80 | 4.6 | 35 | 120 | 2.85 | 32 | 10–103 | 2.9 | 30 | 15–100 | 3.9 | 35 |
| TI [ms] | – | – | - | 2500 | 650 | - | – | 900 | - | – | 900 | - |
| FA [°] | 90 | 30 | 90 | 90 | 8 | 90 | 150 | 9 | 89 | 90 | 8 | 90 |
| ETL | 6 | 1 | - | 1 | 1 | - | 6 | 1 | - | 4 | 128 | 73 |
| TA [min] | 2.43 | 2.75 | 10 | 2.02 | 1.9 | 6.032 | 2.92 | 3.07 | 7 | 8.6 | 8.2 | 10 |
| N vol | - | - | 200 | - | - | 240 | - | - | 140 | - | - | 200 |

**Supplementary Table 2.** Functional connectivity differences in SMN between four groups of people with Multiple Sclerosis and healthy controls.

|  |  |  | MNI coordinates | | |  |
| --- | --- | --- | --- | --- | --- | --- |
| Cluster size (voxels) | **t** | **p** | **x** | **y** | **z** | **Cluster location (local maxima)** |
| LL/LD> HS | | | | | | |
| 519 | 3.47 | 0.0006 | -48 | -18 | 12 | Left Central Opercular Cortex |
|  | 3.45 | 0.0006 | -54 | 4 | 4 | Left Precentral Gyrus |
|  | 3.27 | 0.001 | -40 | -4 | -6 | Left Insular Cortex |
|  | 3.19 | 0.0008 | -54 | 6 | 10 | Left Precentral Gyrus |
| 460 | 3.76 | 0.0004 | -2 | -4 | 56 | Left Supplementary Motor Cortex |
|  | 2.95 | 0.0028 | -6 | -20 | 52 | Left Precentral Gyrus |
|  | 2.01 | 0.0246 | 8 | -8 | 60 | Right Supplementary Motor Cortex |
| 421 | 4.46 | 0.0002 | 36 | -20 | 8 | Right Insular Cortex |
|  | 2.73 | 0.0044 | 60 | -26 | 24 | Right Supramarginal Gyrus (anterior division) |
| 319 | 3.62 | 0.0004 | -36 | -16 | 46 | Left Precentral Gyrus |
|  | 2.38 | 0.0084 | -34 | -26 | 54 | Left Postcentral Gyrus |
| 238 | 2.76 | 0.0036 | 50 | -22 | 44 | Right Postcentral Gyrus |
|  | 2.5 | 0.0056 | 40 | -12 | 44 | Right Precentral Gyrus |
| 163 | 2.94 | 0.0016 | 34 | -26 | 48 | Right Postcentral Gyrus |
|  | 2.23 | 0.0136 | 40 | -20 | 62 | Right Precentral Gyrus |
| HL/LD < HS | | | | | | |
| 283 | -3.3 | 0.0006 | 44 | -6 | 0 | Right Insular Cortex |
|  | -3.2 | 0.001 | 42 | -10 | 12 | Right Central Opercular Cortex |
| LL/HD < HS | | | | | | |
| 224 | -3.3 | 0.0008 | 46 | -24 | 62 | Right Postcentral Gyrus |
|  | -3 | 0.0016 | 54 | -18 | 54 | Right Postcentral Gyrus |
| HL/HD < HS | | | | | | |
| 958 | -3.6 | 0.0004 | 58 | -10 | 48 | Right Postcentral Gyrus |
|  | -3.5 | 0.0004 | 56 | -6 | 48 | Right Precentral Gyrus |
| 551 | -4.2 | 0.0002 | -48 | -12 | 48 | Left Precentral Gyrus |
|  | -2.6 | 0.0054 | -50 | -30 | 50 | Left Postcentral Gyrus |
| 479 | -4.2 | 0.0002 | 0 | -28 | 56 | Right Precentral Gyrus |
|  | -3.2 | 0.0006 | 4 | -12 | 64 | Right Supplementary Motor Cortex |
|  | -2.5 | 0.0072 | 8 | -36 | 50 | Right Precuneous Cortex |
| 343 | -4.7 | 0.0002 | 42 | -2 | 8 | Right Insular Cortex |
| 233 | -3.5 | 0.0002 | -40 | -6 | 12 | Left Central Opercular Cortex |
|  | -2.5 | 0.0096 | -40 | 4 | -12 | Left Insular Cortex |
| 179 | -3.4 | 0.0002 | -22 | -34 | 70 | Left Postcentral Gyrus |
|  | -3.3 | 0.001 | -16 | -22 | 70 | Left Precentral Gyrus |
| 146 | -4.3 | 0.0002 | 62 | 12 | 20 | Right Precentral Gyrus |
|  |  | 0.0008 | 66 | -10 | 32 | Right Postcentral Gyrus |

HS: healthy controls; LL/LD: low lesion volume and low disability; HL/LD: high lesion volume and low disability; LL/HD: low lesion volume and high disability; HL/HD: high lesion volume and high disability. Anatomical localizations of peak Montreal Neurological Institute (MNI) coordinates (mm) were established according to Harvard-Oxford cortical and subcortical structural atlases and the cerebellar atlas included in FMRIB’s Software Library (significance at p<0.05, false discovery rate corrected, minimum cluster extent set at 100 voxels). **Supplementary Table 3.** Functional connectivity differences in DMN between four groups of people with Multiple Sclerosis and healthy controls.

|  |  |  | MNI coordinates | | |  |
| --- | --- | --- | --- | --- | --- | --- |
| Cluster size (voxels) | **t** | **p** | **x** | **y** | **z** | **Cluster location (local maxima)** |
| LL/LD < HS | | | | | | |
| 493 | -3.8 | 0.0002 | -2 | -68 | 46 | Left Precuneous Cortex |
|  | -3.8 | 0.0008 | 6 | -64 | 44 | Right Precuneous Cortex |
|  | -2 | 0.0244 | -2 | -48 | 28 | Left Cingulate Gyrus (posterior division) |
| HL/LD < HS | | | | | | |
| 941 | -5.2 | 0.0002 | 2 | -48 | 34 | Right Cingulate Gyrus (posterior division) |
|  | -5.2 | 0.0002 | 4 | -58 | 36 | Right Precuneous Cortex |
|  | -3.9 | 0.0002 | -2 | -42 | 42 | Left Cingulate Gyrus (posterior division) |
|  | -3.9 | 0.0004 | -2 | -66 | 36 | Left Precuneous Cortex |
| 713 | -4 | 0.0002 | -50 | -62 | 40 | Left Lateral Occipital Cortex (superior division) |
|  | -3.8 | 0.0002 | -46 | -58 | 28 | Left Angular Gyrus |
|  | -2.9 | 0.0024 | -50 | -50 | 24 | Left Supramarginal Gyrus (posterior division) |
| LL/HD < HS | | | | | | |
| 450 | -3.3 | 0.0008 | 14 | -50 | 42 | Right Precuneous Cortex |
|  | -2.8 | 0.0024 | -4 | -46 | 40 | Left Precuneous Cortex |
|  | -2.5 | 0.0076 | -2 | -44 | 30 | Left Cingulate Gyrus (posterior division) |
| 350 | -3.5 | 0.0006 | -40 | -62 | 52 | Left Lateral Occipital Cortex (superior division) |
|  | -3.4 | 0.0008 | -52 | -54 | 34 | Left Angular Gyrus |
| HL/HD < HS | | | | | | |
| 807 | -5.1 | 0.0002 | 0 | -46 | 36 | Left Cingulate Gyrus (posterior division) |
|  | -3.6 | 0.0004 | 4 | -54 | 40 | Right Precuneous Cortex |
|  | -3.4 | 0.0006 | -2 | -68 | 36 | Left Precuneous Cortex |
|  | -2.7 | 0.0036 | -2 | -38 | 36 | Left Cingulate Gyrus (posterior division) |
| 787 | -6.1 | 0.0002 | -54 | -54 | 30 | Left Angular Gyrus |
|  | -5.1 | 0.0002 | -50 | -66 | 40 | Left Lateral Occipital Cortex (superior division) |
|  | -2.9 | 0.0022 | -30 | -56 | 38 | Left Superior Parietal Lobule |
| 181 | -5.3 | 0.0002 | 10 | -54 | -38 | Cerebellar Lobule Right IX |
|  | -4.5 | 0.0002 | -2 | -60 | -46 | Left Vermis IX |
|  | -4.2 | 0.0002 | -8 | -56 | -38 | Cerebellar Lobule Left IX |
| 125 | -4.2 | 0.0002 | 56 | -62 | 34 | Right Lateral Occipital Cortex (superior division) |
|  | -3.6 | 0.0006 | 52 | -52 | 30 | Right Angular Gyrus |
| 105 | -3.8 | 0.0004 | -26 | 18 | 56 | Left Superior Frontal Gyrus |

HS: healthy controls; LL/LD: low lesion volume and low disability; HL/LD: high lesion volume and low disability; LL/HD: low lesion volume and high disability; HL/HD: high lesion volume and high disability. Anatomical localizations of peak Montreal Neurological Institute (MNI) coordinates (mm) were established according to Harvard-Oxford cortical and subcortical structural atlases and the cerebellar atlas included in FMRIB’s Software Library (significance at p<0.05, false discovery rate corrected, minimum cluster extent set at 100 voxels).

**Supplementary Table 4.** Functional connectivity differences in SMN between groups of people with Multiple Sclerosis.

|  |  |  | MNI coordinates | | |  |
| --- | --- | --- | --- | --- | --- | --- |
| Cluster size (voxels) | **t** | **p** | **x** | **y** | **z** | **Cluster location (local maxima)** |
| LL/LD > HL/LD | | | | | | |
| 841 | 4.55 | 0.0002 | 42 | -6 | -4 | Right Insular Cortex |
|  | 3.98 | 0.0002 | 40 | -14 | 14 | Right Central Opercular Cortex |
| 587 | 3.78 | 0.0004 | -44 | -6 | -4 | Left Insular Cortex |
|  | 2.7 | 0.0044 | -44 | -16 | 12 | Left Central Opercular Cortex |
|  | 2.66 | 0.0044 | -56 | 8 | 14 | Left Precentral Gyrus |
| 433 | 3.66 | 0.0002 | -2 | -4 | 58 | Left Supplementary Motor Cortex |
|  | 2.71 | 0.0040 | 12 | -36 | 56 | Right Postcentral Gyrus |
|  | 2.12 | 0.0142 | 2 | -20 | 52 | Right Precentral Gyrus |
| 283 | 3.27 | 0.0004 | 26 | -30 | 60 | Right Postcentral Gyrus |
| 277 | 2.62 | 0.0056 | 58 | -16 | 42 | Right Postcentral Gyrus |
|  | 2.09 | 0.0190 | 50 | -2 | 50 | Right Precentral Gyrus |
| 245 | 3.49 | 0.0008 | -46 | -10 | 50 | Left Precentral Gyrus |
|  | 2.72 | 0.0036 | -40 | -20 | 46 | Left Postcentral Gyrus |
| LL/HD > HL/HD | | | | | | |
| 228 | 3.19 | 0.0006 | 4 | -4 | 56 | Right Supplementary Motor Cortex |
|  | 2.35 | 0.0100 | 0 | -16 | 68 | Right Precentral Gyrus |
| 153 | 3.03 | 0.0016 | -40 | -16 | 4 | Left Insular Cortex |
|  | 2.21 | 0.0146 | -48 | -16 | 14 | Left Central Opercular Cortex |
| 115 | 2.79 | 0.0028 | 24 | -24 | 70 | Right Precentral Gyrus |
|  | 2.19 | 0.0122 | 30 | -36 | 66 | Right Postcentral Gyrus |
| 110 | 2.53 | 0.0052 | 52 | -8 | 52 | Right Precentral Gyrus |
|  | 2.37 | 0.0074 | 50 | -12 | 56 | Right Postcentral Gyrus |
| LL/LD > LL/HD | | | | | | |
| 713 | 2.67 | 0.0036 | -42 | -6 | 58 | Left Precentral Gyrus |
|  | 2.69 | 0.0056 | -50 | -24 | 54 | Left Postcentral Gyrus |
| 712 | 4.41 | 0.0002 | 46 | -26 | 62 | Right Postcentral Gyrus |
|  | 3.58 | 0.0004 | 38 | -16 | 44 | Right Precentral Gyrus |
| 469 | 2.84 | 0.0022 | 42 | 6 | -4 | Left Insular Cortex |
|  | 3.09 | 0.0018 | 46 | -4 | 2 | Right Central Opercular Cortex |
| 391 | 3.07 | 0.0014 | -8 | -16 | 48 | Left Precentral Gyrus |
|  | 2.64 | 0.0044 | -2 | -4 | 58 | Left Supplementary Motor Cortex |
|  | 2.26 | 0.0126 | 4 | -28 | 54 | Right Precentral Gyrus |
| 320 | 3.13 | 0.0014 | -40 | -16 | -2 | Left Insular Cortex |
| HL/LD> HL/HD | | | | | | |
| 576 | 3.32 | 0.0006 | -44 | -16 | 58 | Left Precentral Gyrus |
|  | 2.01 | 0.0216 | -48 | -32 | 50 | Left Postcentral Gyrus |
| 471 | 3.09 | 0.0016 | 46 | -6 | 56 | Right Precentral Gyrus |
|  | 2.54 | 0.0046 | 52 | -20 | 56 | Right Postcentral Gyrus |
| 256 | 2.86 | 0.0022 | 0 | -24 | 54 | Right Precentral Gyrus |
|  | 2.24 | 0.0124 | 12 | -12 | 44 | Right Supplementary Motor Cortex |
| HL/LD > LL/HD | | | | | | |
| 161 | 3.25 | 0.0010 | -34 | -16 | 68 | Left Precentral Gyrus |
| 100 | 2.39 | 0.0106 | 52 | -20 | 56 | Right Postcentral Gyrus |
|  | 2.41 | 0.0094 | 40 | -16 | 50 | Right Precentral Gyrus |

LL/LD: low lesion volume and low disability; HL/LD: high lesion volume and low disability; LL/HD: low lesion volume and high disability; HL/HD: high lesion volume and high disability. Anatomical localizations of peak Montreal Neurological Institute (MNI) coordinates (mm) were established according to Harvard-Oxford cortical and subcortical structural atlases and the cerebellar atlas included in FMRIB’s Software Library (significance at p<0.05, false discovery rate corrected, minimum cluster extent set at 100 voxels).

**Supplementary Table 5.** Functional connectivity differences in DMN between groups of people with Multiple Sclerosis.

|  |  |  | MNI coordinates | | |  |
| --- | --- | --- | --- | --- | --- | --- |
| Cluster size (voxels) | **t** | **p** | **x** | **y** | **z** | **Cluster location (local maxima)** |
| LL/LD > HL/LD | | | | | | |
| 514 | 4.46 | 0.0002 | 2 | -48 | 34 | Right Cingulate Gyrus (posterior division) |
|  | 4.27 | 0.0002 | -2 | -42 | 42 | Left Cingulate Gyrus (posterior division) |
|  | 3.37 | 0.0006 | 4 | -58 | 36 | Right Precuneous Cortex |
|  | 2.78 | 0.0028 | -10 | -50 | 46 | Left Precuneous Cortex |
| 476 | 3.66 | 0.0004 | -52 | -60 | 40 | Left Lateral Occipital Cortex (superior division) |
|  | 3.04 | 0.0024 | -48 | -54 | 40 | Left Angular Gyrus |
|  | 2.09 | 0.0168 | -50 | -50 | 24 | Left Supramarginal Gyrus (posterior division) |
| LL/HD > HL/HD | | | | | | |
| 282 | 2.94 | 0.0006 | -50 | -68 | 40 | Left Lateral Occipital Cortex (superior division) |
|  | 3.14 | 0.0004 | 4 | -54 | -46 | Cerebellar Lobule Right IX |
|  | 2.56 | 0.0060 | -2 | -58 | -46 | Cerebellar Lobule Left IX |
| HL/LD > HL/HD | | | | | | |
| 166 | 3.29 | 0.0004 | 4 | -44 | 26 | Right Cingulate Gyrus (posterior division) |
|  | 3.06 | 0.0010 | 0 | -56 | 16 | Right Precuneous Cortex |
|  | 2.92 | 0.0016 | -2 | -44 | 26 | Left Cingulate Gyrus (posterior division) |
|  | 2.6 | 0.0054 | 6 | -60 | 8 | Right Lingual Gyrus |
| 132 | 3.49 | 0.0004 | 4 | -58 | 36 | Right Precuneous Cortex |
| LL/HD > HL/LD | | | | | | |
| 328 | 3.5 | 0.0006 | 4 | -48 | 34 | Right Cingulate Gyrus (posterior division) |
|  | 3.05 | 0.0010 | 0 | -62 | 8 | Right Lingual Gyrus |
|  | 2.8 | 0.0026 | 8 | -60 | 10 | Right Precuneous Cortex |
|  | 2.79 | 0.0040 | -2 | -40 | 42 | Left Cingulate Gyrus (posterior division) |

LL/LD: low lesion volume and low disability; HL/LD: high lesion volume and low disability; LL/HD: low lesion volume and high disability; HL/HD: high lesion volume and high disability. Anatomical localizations of peak Montreal Neurological Institute (MNI) coordinates (mm) were established according to Harvard-Oxford cortical and subcortical structural atlases and the cerebellar atlas included in FMRIB’s Software Library (significance at p<0.05, false discovery rate corrected, minimum cluster extent set at 100 voxels).

**Supplementary Table 6.A.** Percentage of PwMS in the four groups that scored less than the cutoff (Amato et al. 2006) at education-corrected Paced Auditory Serial Addition Test with 3.0-seconds interstimulus interval (PASAT3), Symbol Digit Modalities Test (SDMT) and at one or both the tests.

|  | **HS** | **LL/LD** | **HL/LD** | **LL/HD** | **HL/HD** |
| --- | --- | --- | --- | --- | --- |
| **PASAT3 and/or SDMT** | 10%  (8/81) | 18%  (29/165) | **30%**  **(27/91)***** | 16%  (10/64) | **45%**  **(56/124)***** |
| **PASAT3** | 3%  (5/147) | **13%**  **(26/207)***** | **17%**  **(20/118)***** | 11%  (11/100) | **25%**  **(41/166)***** |
| **SDMT** | 6%  (5/82) | 7%  (12/172) | 16%  (15/95) | 11%  (8/70) | **41%**  **(55/135)***** |

Differences with HS are calculated via Chi-square test and significance is reported in bold font. ***(p < 0.001), ** (p<0.01), and * (p<0.05)

**Supplementary Table 6.B. Chi-square between groups.**

|  | **PASAT3 and/or SDMT** | | **PASAT3** | | **SDMT** | |
| --- | --- | --- | --- | --- | --- | --- |
|  | **Chi-square** | **p-value** | **Chi-square** | **p-value** | **Chi-square** | **p-value** |
| **LL/LD vs HL/LD** | **5.02** | **0.03** | 1.23 | 0.32 | **5.31** | **0.02** |
| **LL/LD vs LL/HD** | 0.12 | 0.07 | 0.12 | 0.85 | 1.41 | 0.27 |
| **LL/LD vs HL/HD** | **25.95** | **<0.001** | **9.34** | **0.003** | **50.91** | **<0.001** |
| **HL/LD vs LL/HD** | **4.08** | **0.04** | 1.50 | 0.26 | 0.58 | 0.52 |
| **HL/LD vs HL/HD** | 5.31 | 0.21 | 2.46 | 0.13 | **16.40** | **<0.001** |
| **LL/HD vs HL/HD** | **16.17** | **<0.001** | **7.26** | **0.008** | **18.17** | **<0.001** |

**Supplementary Table 7.** Structural Magnetic Resonance Imaging measures of the subgroup of people with Multiple Sclerosis HL/HD and lesion volume comparable to that of HL/LD.

| **N** | **LV** | **GM** | **WM** | **Thalamus** | **Caudate** | **Putamen** | **Pallidum** | **Sensorimotor**  **cerebellum** | **Cognitive**  **cerebellum** | **C2-C3 area [cm2]** |
| --- | --- | --- | --- | --- | --- | --- | --- | --- | --- | --- |
| 189 | 12.43 (5.84) | 37.40 (4.17) | 38.94 (4.37) | 0.86 (0.11) | 0.41 (0.05) | 0.54 (0.08) | 0.20 (0.04) | 2.27 (0.24) | 5.52 (0.55) | 0.55 (0.09) |

HL/HD: high lesion volume and high disability; HL/LD: high lesion volume and low disability. Values are reported as average (standard deviation). Brain structure volumes are reported as a percentage of the Total Intracranial Volume (TIV). Subcortical volumes are considered as the sum of the right and left.
